# Repeatability and agreement of biometric measurements using spectral domain anterior segment optical coherence tomography and Scheimpflug tomography in keratoconus

**DOI:** 10.1371/journal.pone.0248659

**Published:** 2021-05-21

**Authors:** Ye Li, Akilesh Gokul, Charles McGhee, Mohammed Ziaei

**Affiliations:** Department of Ophthalmology, New Zealand National Eye Centre, Faculty of Medical and Health Sciences, University of Auckland, Auckland, New Zealand; Universidad de Monterrey Division de Ciencias de la Salud, MEXICO

## Abstract

**Purpose:**

To compare the repeatability and agreement in biometric measurements using Spectral Domain Anterior Segment OCT (AS-OCT, REVO-NX, Optopol) and Scheimpflug tomography (Pentacam-AXL, Oculus) in keratoconus.

**Methods:**

Prospective case series at a university hospital tertiary center. Axial length (AL), anterior chamber depth (ACD), central corneal thickness (CCT), and thinnest corneal thickness (TCT) were measured using both devices in patients with keratoconus. Three groups were analyzed: eyes with no prior crosslinking or contact lens wear (Group A), eyes with prior crosslinking (Group B), and eyes with prior contact lens wear (Group C). Repeatability and agreement of measurements were analyzed.

**Results:**

The study comprised of 214 eyes of 157 subjects. In Group A (n = 95 eyes), Group B (n = 86 eyes), and Group C (n = 33 eyes), intraclass correlation coefficient (ICC) was higher than 0.90 for all examined parameters, except for ACD readings in Group A with the REVO-NX (ICC = 0.83). Differences in ACD, TCT, and CCT were significantly different between the two devices for Groups A, B and C (p<0.05). AL measurements differed significantly in Groups A and B (p<0.05) but not in Group C (p = 0.18). Repeatability did not vary significantly between Groups A, B, or C in any parameter with both devices (p>0.05). There was poor agreement between the two devices across all parameters (p<0.05).

**Conclusions:**

Both devices demonstrated good repeatability but poor agreement across AL, ACD, CCT and TCT measurements. There was no significant difference in repeatability in virgin eyes compared to eyes with prior crosslinking or contact lens wear, however, the interchangeable use of the two devices is not recommended.

## Introduction

Keratoconus is the most common form of corneal ectasia characterized by corneal steepening and irregular astigmatism [1, 2], and is typically treated in a staged manner with refractive correction, cornea crosslinking (CXL) or corneal transplantation [3–7]. Patients with keratoconus are at an increased risk of cataract formation due to associated atopy and steroid use [8]. Such patients present unique challenges for the cataract surgeon regarding intraocular lens (IOL) power calculation. The inherent difficulty in obtaining accurate biometric measurements, changes in the relationship between the anterior and posterior cornea, inaccurate calculation of the effective lens position due to inaccurate keratometry measurement, and axis of astigmatism [9] can result in unpredictable refractive outcomes [10]. Reduction of biometric measurement error can therefore optimize post-operative refractive and visual outcomes in patients with keratoconus.

Accurate biometric measurements can be challenging in patients with keratoconus, and multiple publications have reported differences in biometric measurements obtained from different devices [11–14], including anterior chamber depth (ACD), keratometry, central corneal thickness (CCT), thinnest corneal thickness (TCT), axial length (AL), and lens thickness (LT).

REVO-NX anterior segment spectral-domain optical coherence tomography (AS-OCT) (Optopol Technology S.A) and Pentacam-AXL Scheimpflug imaging system (Oculus Optikgeräte GmbH) are recently introduced contact-free devices which can measure ocular biometric parameters. REVO-NX combines AS-OCT with optical biometry to generate ocular cross-sections using low-coherence interferometry [15]. Pentacam-AXL utilizes a rotating Scheimpflug imaging system and incorporates partial coherence interferometry to obtain AL measurements [16]. This study aimed to assess the repeatability and agreement of biometric measurements obtained by REVO-NX and Pentacam-AXL in keratoconic patients with or without a history of CXL or contact lens use.

## Methods

This prospective study enrolled patients with keratoconus attending the University of Auckland Cornea and External Eye Disease Service, Auckland District Health Board, Auckland, New Zealand from January to August 2019.

Patients who were diagnosed with keratoconus based on clinical and topographic features were included [17, 18]. For keratoconus diagnosis and classification, we analyzed the topographic sagittal curvature pattern, posterior and anterior elevation maps and corneal thickness pattern, in addition to information from the Belin-Ambrosio Enhanced Ectasia Display. Diagnosis was confirmed using the inbuilt parameters of the Pentacam including a Keratoconus Index (KI, ≥ 1.07) and Topographic Keratoconus Classification (TKC ≥ 1) [19]. Keratoconus severity was staged according to the TKC from 0 (normal) to 4 (severe) [20].

Exclusion criteria included corneal scarring, edema, severe atopy, dry eye, blepharitis, trauma, or prior ocular surgery other than CXL. Contact lens wearers of any type were instructed to remove their contact lenses at least 48 hours prior to the exam. Patients were analyzed in three groups: patients with no prior CXL or contact lens wear (Group A), patients with a history of previous CXL between 3 to 6-months prior (Group B), and patients with prior contact lens wear of any type, including soft, rigid gas permeable (RGP), semi-scleral, piggyback, and hybrid lenses (Group C). Only one eye from each individual was used for analysis within each group. The right eye of each patient was the default choice for analysis, but the left eye was used if any of the exclusion criteria applied. The only situation where both eyes of one patient were included was if one eye had a history of crosslinking or contact lens wear and the other was an eye with no previous intervention, but eyes from the same patient were never analyzed together in the same group. For comparison of repeatability between disease severities, those with disease severity between categories were rounded up (e.g. Stage 1–2 = Stage 2).

The study was approved by the Health and Disability Ethics Committee, a branch of the Ministry of Health in New Zealand. Written, informed consent was obtained from all patients after they voiced understanding of the purpose and the procedures of the study in accordance with the Declaration of Helsinki.

### Instruments

The REVO-NX is an optical biometer combined with anterior and posterior segment SD-OCT which uses an 830nm super-luminescent laser diode to measure biometric parameters as an average of 10 B-scans. This device obtains 110,000 scans per second at a scan depth of 2.4mm, axial resolution of 5μm, and transverse resolution of 12μm [15].

The Pentacam-AXL is a partial coherence interferometry device that combines a rotating Scheimpflug system with optical biometry using a blue 475nm light-emitting diode. The device acquires 25-images per scan to produce high-resolution corneal measurements [16]. The presence of a second camera detects and corrects for any eye movement. Three-dimensional Scheimpflug images are created with a central fine-meshed dot matrix. Anterior corneal surface images derived over a 3-mm diameter are used for the calculation of simulated keratometry values [16].

### Patient assessment

All patients received a thorough ocular assessment. Both devices were calibrated and eyes were scanned three consecutive times on each device by one of two experienced examiners, in a random order. All measurements were performed without pupil dilation and under identical lighting conditions between 1.00 pm and 5.00 pm to limit the influence of overnight corneal swelling [21]. Subjects were asked to fixate on the target and blink immediately before each measurement to enable adequate tear film coverage. Scans of acceptable quality were included as indicated by “ok” for Pentacam-AXL and a quality score greater than 5 for REVO-NX. Automatic capture was enabled for both devices to eliminate differences between scans captured by the two different investigators.

### Statistical analysis

Statistical analysis was performed using SPSS (SPSS, IBM, Chicago, Illinois, USA). Kolmogorov-Smirnov test assessed for normality of distribution. Within-subject standard deviation (Sw) was used to calculate precision (1.96xSw) and repeatability (2.77xSw). Repeatability of the devices was assessed through the coefficient of variation (CV) and intraclass correlation coefficient (ICC).

Bland-Altman plots were used to assess agreement between the two devices [22]. When the mean difference was statistically significant (fixed bias), linear regression was used to assess for proportional bias. The 95% limits of agreement (LoA) was calculated through mean difference ± 1.96 x standard deviation, which indicates the range where most of the mean differences in measurement are situated.

Using Sw, One-way ANOVA with Tukey’s post-hoc assessed for differences in repeatability between the three groups and between different keratoconus grades. Pearson correlation coefficients between repeatability and maximum keratometry (K_MAX_) were calculated. A *p* value of <0.05 was deemed significant.

### Sample size calculation

Considering the novelty of the REVO-NX and its minimal published biometry investigations, sample size calculations were performed based on the recent investigation of the device on AL in normal subjects by Kanclerz *et al* [15]. A minimum of 47 eyes was required to produce a similar level of repeatability at a significance level of 0.05, power of 80% and standard deviation of 1.07 [15]. A minimum of 52 eyes was required if the REVO-NX and Pentacam AXL have a similar agreement in biometric parameters as the REVO-NX and Lenstar at a significance level of 0.05, power of 80% and standard deviation of 0.02mm [15].

## Results

### Demographics

The study comprised of 214 eyes of 157 patients. 95 eyes of 95 patients were included in Group A, 86 eyes of 86 patients were included in Group B, and 33 eyes of 33 patients were included in Group C. Table 1 describes the demographic details of the patients included in the study.

**Table 1:** Demographic information of all patients included in the study.

Parameters		Value
Patients (n)		157
Eyes (n)		214
	Right	149 (69.6%)
	Left	65 (30.4%)
Age (mean ± SD, range)	All patients	24.50±7.69, 10–64
Virgin Eyes		
	No intervention	95 (44.3%)
CXL		
	Yes	86 (40.2%)
	No	128 (59.8%)
Contact lens use		
	No contact lens	181 (84.6%)
	RGP	10 (4.7%)
	Semi-scleral	6 (2.8%)
	Soft	12(5.6%)
	Piggyback	3 (1.4%)
	Hybrid	2 (0.9%)
Keratoconus stage (TKC)		
	1	43 (20.1%)
	1–2	17 (7.9%)
	2	38 (17.8%)
	2–3	22 (10.3%)
	3	66 (30.8%)
	3–4	28 (13.1%)
K_MAX_ (Mean ± SD, D)	All patients	56.17± 8.08
	Virgin eyes	54.19 ± 7.80
	Prior CXL	57.55 ± 7.51
	Contact lens use	58.33± 9.20

CXL, crosslinking; RGP, rigid gas permeable; TKC, topographic keratoconus classification; K_MAX_, maximum keratometry; D, diopter.

### Repeatability of biometric measurements

In Group A, aside from an ICC of 0.83 for ACD in REVO-NX, ICC was above 0.97 for all other parameters for both devices. In Group B, ICC was greater than 0.97 for both devices. In Group C, ICC was greater than 0.99 in ACD, CCT, TCT for both devices. In all groups, ICC was higher in Pentacam-AXL for all parameters.

The precision, repeatability, CV, and ICC of biometric parameters are displayed in Table 2.

**Table 2:** S_w_, precision, repeatability, CV, and ICC (95% confidence interval).

Parameter (units)	Mean ± SD	Within-Subject SD	Precision	Repeatability	CV (%)	ICC	ICC 95% Confidence Interval
**GROUP A (n = 95)**							
ACD (mm)							
• REVO NX	3.75 ± 0.31	0.08	0.16	0.22	0.82	0.83	0.75 to 0.88
• Pentacam-AXL	3.83 ± 0.31	0.03	0.05	0.08	0.40	0.99	0.99 to 1.00
CCT (μm)							
• REVO NX	480.28 ± 42.25	10.73	21.03	29.72	1.59	0.98	to 0.99
• Pentacam-AXL	474.89 ± 42.44	2.92	5.73	8.10	0.43	0.99	0.99 to 1.00
AL (mm)							
• REVO NX	24.02 ± 0.85	0.13	0.25	0.35	0.09	0.99	0.99 to 1.00
• Pentacam-AXL	23.97 ± 0.83	0.01	0.03	0.04	0.00	1.00	1.00 to 1.00
TCT (μm)							
• REVO NX	440.19 ± 46.54	13.25	25.96	36.69	1.01	0.97	0.96 to 0.98
• Pentacam-AXL	464.84± 44.65	2.98	8.26	0.44	0.44	0.99	0.99 to 0.99
LT (mm)							
• REVO NX	4.89 ± 12.64	0.08	0.16	0.22	1.28	0.98	0.97 to 0.98
**GROUP B (n = 86)**							
ACD (mm)							
• REVO NX	3.80±0.33	0.10	0.19	0.27	1.08	0.97	0.96 to 0.98
• Pentacam-AXL	3.89±0.35	0.03	0.06	0.08	0.43	0.99	0.99 to 1.00
CCT (μm)							
• REVO NX	466.44±43.66	9.65	18.91	26.72	1.35	0.98	0.98 to 0.99
• Pentacam-AXL	456.26±45.18	3.70	7.26	10.26	0.49	0.99	0.99 to 1.00
AL(mm)							
• REVO NX	23.91±0.95	0.08	0.16	0.23	0.05	0.99	0.99 to 1.00
• Pentacam-AXL	23.89±0.96	0.02	0.04	0.06	0.00	1.00	1.00 to 1.00
TCT (μm)							
• REVO NX	424.99 ± 43.47	5.18	10.15	14.35	0.56	0.99	0.99 to 1.00
• Pentacam-AXL	444.81 ± 45.13	3.48	6.82	9.63	0.62	0.99	0.99 to 1.00
LT (mm)							
• REVO NX	3.57±0.27	0.15	0.29	0.42	1.52	0.90	0.85 to 0.93
**GROUP C (n = 33)**							
ACD (mm)							
• REVO NX	3.70±0.38	0.03	0.06	0.09	0.70	0.99	0.99 to 1.00
• Pentacam-AXL	3.79±0.39	0.03	0.05	0.07	0.39	0.99	0.99 to 1.00
CCT(μm)							
• REVO NX	450.26±43.04	6.49	12.71	17.97	1.03	0.99	0.99 to 1.00
• Pentacam-AXL	444.43±47.89	4.48	8.77	12.40	0.58	0.99	0.99 to 1.00
AL (mm)							
• REVO NX	23.95±0.98	0.10	0.20	0.28	0.06	0.99	0.99 to 1.00
• Pentacam-AXL	23.92±0.97	0.01	0.03	0.04	0.00	1.00	1.00 to 1.00
TCT (μm)							
• REVO NX	416.33±45.59	3.25	6.37	9.00	0.27	0.99	0.99 to 1.00
• Pentacam-AXL	435.17±45.28	4.33	8.49	11.99	0.61	0.99	0.99 to 1.00
LT (mm)							
• REVO NX	3.61±0.33	0.06	0.12	0.17	1.21	0.99	0.98 to 0.99

CV, coefficient of variation; ICC, intraclass correlation coefficient; ACD, anterior chamber depth; CCT, central corneal thickness; AL, axial length; TCT, thinnest corneal thickness; LT, lens thickness.

### Agreement of biometric measurements

**Fig 1:**
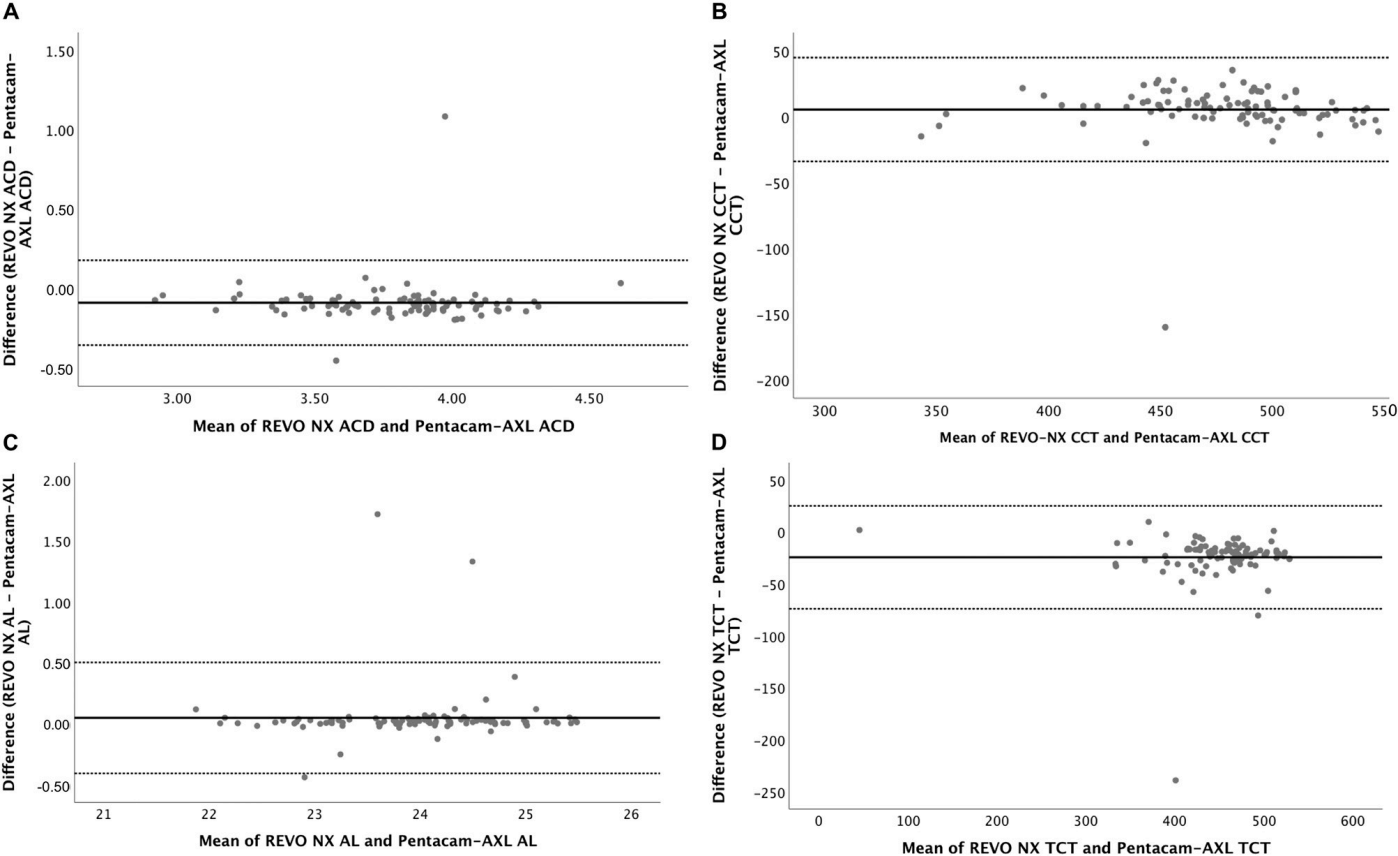
Bland-Altman plots for ACD (1A), CCT (1B), AL (1C), and TCT (1D) for REVO-NX and Pentacam-AXL in Group A eyes. ACD, anterior chamber depth; CCT, central corneal thickness; AL, axial length; TCT, thinnest corneal thickness.

**Fig 2:**
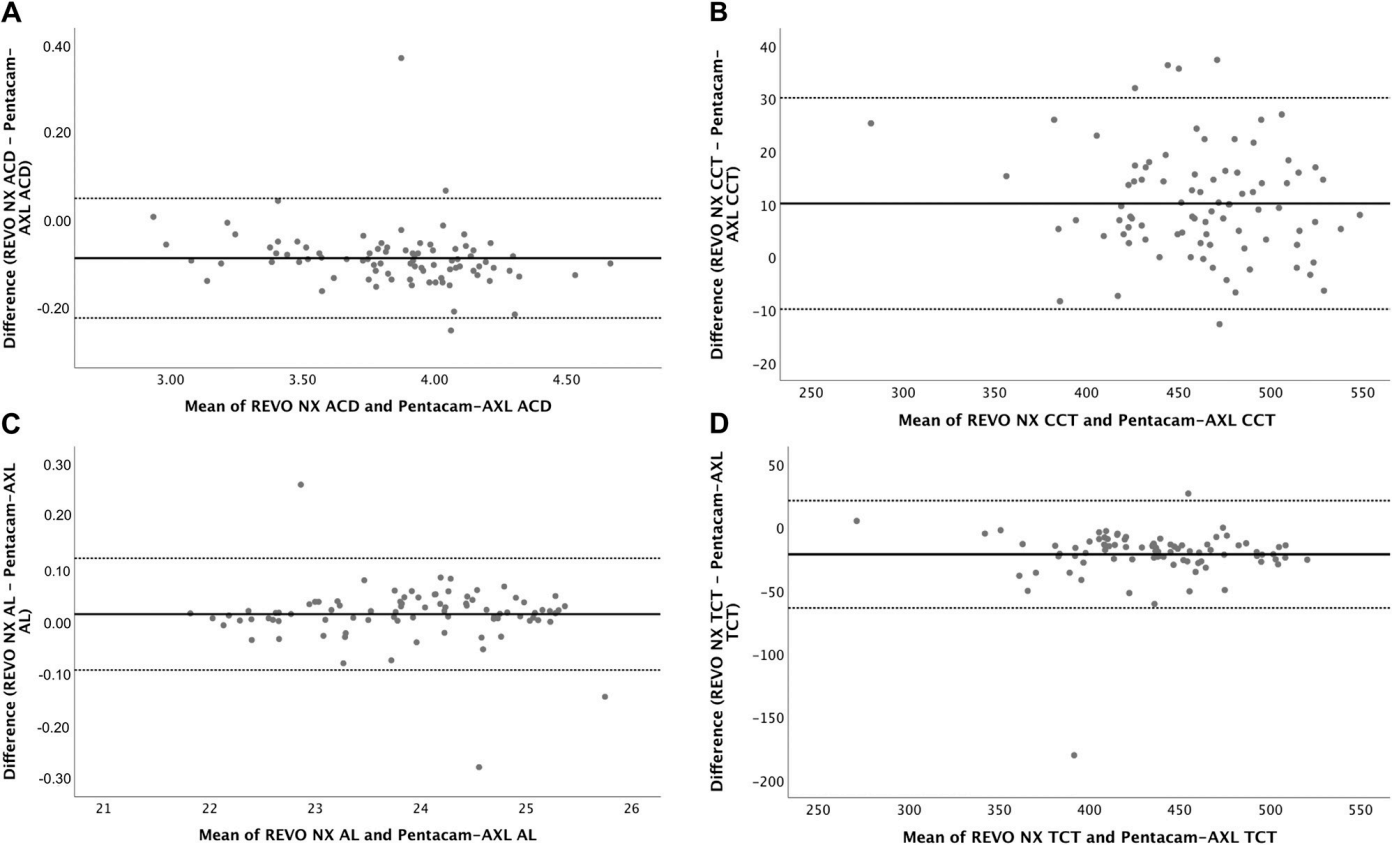
Bland-Altman plots for ACD (2A), CCT (2B), AL (2C), and TCT (2D) for REVO-NX and Pentacam-AXL in Group B eyes. ACD, anterior chamber depth; CCT, central corneal thickness; AL, axial length; TCT, thinnest corneal thickness.

**Fig 3:**
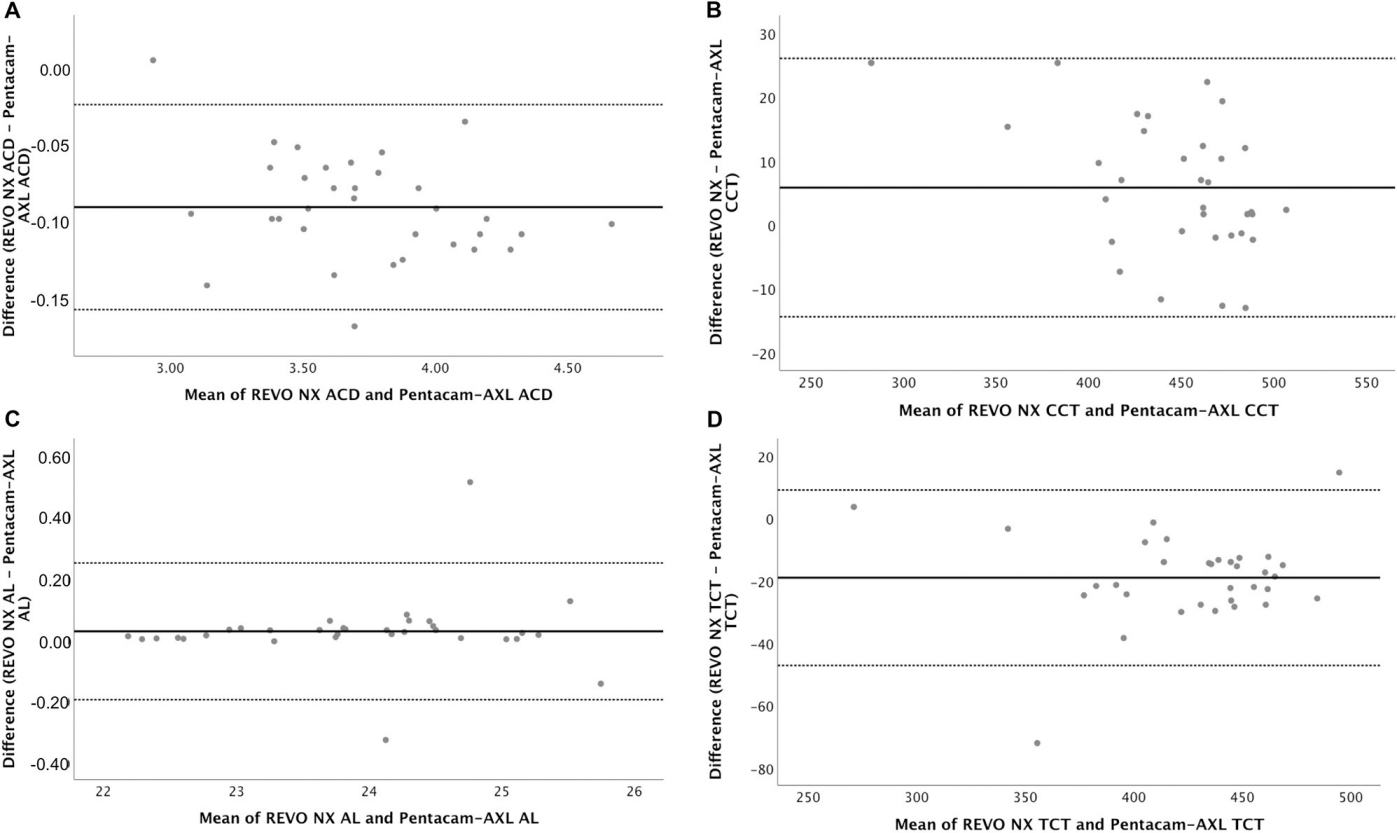
Bland-Altman plots for ACD (3A), CCT (3B), AL (3C), and TCT (3D) for REVO-NX and Pentacam-AXL in Group C eyes. ACD, anterior chamber depth; CCT, central corneal thickness; AL, axial length; TCT, thinnest corneal thickness.

Bland-Altman plots for ACD, CCT, TCT and AL are displayed in Figs 1–3 for Groups A, B, and C respectively. Table 3 shows the mean difference in measurements and the 95% LoA between the two devices.

**Table 3:** Agreement between REVO-NX and Pentacam-AXL in biometric measurements.

Subgroup	Parameter (units)	Mean Difference	*p* Value	Fixed Bias	Proportional Bias	95% LoA
**Group A**	ACD (mm)	-0.09 ± 0.14	<0.01	Yes	No	-0.35 to 0.18
	CCT (μm)	5.39 ± 20.10	0.01	Yes	No	-34.00 to 44.79
	AL (mm)	0.05±0.23	0.03	Yes	No	-0.40 to 0.51
	TCT (μm)	24.38±25.27	<0.01	Yes	No	-73.90 to 25.15
**Group B**	ACD (mm)	-0.09±0.07	<0.01	Yes	Yes	-0.23 to 0.05
	CCT (μm)	10.17±10.20	<0.01	Yes	No	-9.82 to 30.17
	AL (mm)	0.01±0.05	0.02	Yes	No	-0.09 to 0.12
	TCT (μm)	-21.42±21.66	<0.01	Yes	No	-63.88 to 21.04
**Group C**	ACD (mm)	-0.09±0.03	<0.01	Yes	No	-0.16 to -0.02
	CCT (μm)	5.83±10.31	<0.01	Yes	Yes	-14.38 to 26.04
	AL (mm)	0.03±0.11	0.18	No	No	-0.20 to 0.25
	TCT (μm)	-19.00±14.35	<0.01	Yes	No	-47.12 to 9.12

LoA, limits of agreement; ACD, anterior chamber depth; CCT, central corneal thickness; AL, axial length; TCT, thinnest corneal thickness; LT, lens thickness.

In Group A and Group B, all measured parameters were significantly different between REVO-NX and Pentacam-AXL (*p*<0.05). In Group C, with the exception of AL (*p* = 0.18), all other parameters were significantly different (*p*<0.05).

### Comparison of repeatability between three groups

There was no statistically significant difference in repeatability in any measurements between the three groups in pairwise comparisons (*p*>0.05) (Table 4).

**Table 4:** Comparison of repeatability between groups.

Parameter (units)	Device	Comparison (group)	Mean Difference	Standard Error	p value	95% Confidence Interval
ACD (mm)	REVO NX	A vs B	<-0.01	0.01	0.99	-0.03 to 0.02
		A vs C	0.02	0.02	0.49	-0.02 to 0.05
		B vs C	0.02	0.01	0.46	-0.02 to 0.05
	Pentacam-AXL	A vs B	<-0.01	<0.01	0.92	-0.01 to 0.01
		A vs C	<0.01	<0.01	0.92	0.00 to 0.01
		B vs C	<0.01	<0.01	0.78	-0.01 to 0.01
CCT (μm)	REVO NX	A vs B	1.01	1.05	0.60	-1.46 to 3.49
		A vs C	2.20	1.42	0.27	-1.16 to 5.56
		B vs C	1.19	1.44	0.69	-2.22 to 4.59
	Pentacam-AXL	A vs B	-0.32	0.35	0.64	-1.14 to 0.51
		A vs C	-0.07	0.47	0.30	-1.82 to 0.41
		B vs C	-0.39	0.48	0.70	-1.51 to 0.74
AL(mm)	REVO NX	A vs B	0.02	0.02	0.58	-0.02 to 0.05
		A vs C	0.01	0.02	0.84	-0.04 to 0.06
		B vs C	<-0.01	0.02	0.98	-0.05 to 0.05
	Pentacam-AXL	A vs B	<-0.01	<0.01	0.56	-0.01 to 0.00
		A vs C	<0.01	<0.01	0.99	-0.01 to 0.01
		B vs C	<0.01	<0.01	0.67	0.00 to 0.01
TCT (μm)	REVO NX	A vs B	2.03	1.30	0.27	-1.07 to 5.13
		A vs C	3.03	1.78	0.21	-1.18 to 7.24
		B vs C	1.00	1.81	0.85	-3.27 to 5.27
	Pentacam-AXL	A vs B	-0.44	0.31	0.33	-1.18 to 0.29
		A vs C	-0.58	0.42	0.35	-1.57 to 0.41
		B vs C	-0.14	0.43	0.94	-1.14 to 0.87

ACD, anterior chamber depth; CCT, central corneal thickness; AL, axial length; TCT, thinnest corneal thickness.

### Comparison of repeatability between different keratoconus stages

In Groups A and C, there were no statistically significant differences in repeatability between different stages of keratoconus in any of the parameters by either device.

In Group B, there was a statistically significant difference in repeatability in REVO-NX derived AL measurements (*p*<0.01), where stage I eyes had higher variation than stage II (mean difference = 0.10, *p*<0.01), stage III (mean difference = 0.101, *p*<0.01), and stage IV (mean difference = 0.102, *p*<0.01). There was no statistically significant difference in repeatability of any Pentacam-AXL derived measurements or REVO-NX derived ACD, CCT, and TCT measurements between different disease severities.

### Correlation between repeatability and K_MAX_

Pentacam-AXL derived K_MAX_ was correlated with both Pentacam-AXL and REVO-NX derived biometric measurement variation.

In Group A, there was a positive correlation between K_MAX_ and REVO-NX derived CCT variation (*r* = 0.30, *p*<0.01), and a positive correlation between K_MAX_ and Pentacam-AXL derived AL variation (*r* = 0.40, *p*<0.01).

In Group B, there was a negative correlation between K_MAX_ and REVO-NX derived AL variation (*r* = -0.22, *p* = 0.04), and a positive correlation between K_MAX_ and Pentacam-AXL derived CCT (*r* = 0.28, *p* = 0.01), AL (*r* = 0.25, *p* = 0.02), and CCT variations (*r* = 0.24, *p* = 0.03).

In Group C, there was a positive correlation between K_MAX_ and Pentacam-AXL derived CCT variation (*r* = 0.40, *p* = 0.02) and TCT variation (*r* = 0.37, *p* = 0.03). No statistically significant correlations between K_MAX_ and REVO-NX measurement variation were found.

## Discussion

There is an abundance of devices capable of biometric measurements. Investigation of the agreement between various devices determines the interchangeability of use in patient assessment, management, and long-term surveillance in various clinical scenarios including cataract surgery.

Previous studies have demonstrated that in normal eyes, AS-OCT and Pentacam Scheimpflug imaging can provide repeatable measurements for AL, ACD, CCT, and LT using various biometers [15, 23, 24]. There is, however, a paucity of studies focusing on patients with keratoconus, where accurate measurements can be particularly difficult due to a longer AL, longer posterior segment, and deeper ACD [25].

To the best of our knowledge, this is the first report describing the repeatability and agreement of biometric measurements using REVO-NX and Pentacam-AXL in keratoconus. Understanding the differences in measurement parameters between devices is instrumental in optimizing the accuracy of IOL calculations and subsequently refractive outcomes, in patients with keratoconus who are at an increased risk of developing visually significant cataracts [8]. The results of this study demonstrate high repeatability but poor agreement in the biometric parameters as measured by REVO-NX AS-OCT and Pentacam-AXL in patients with keratoconus, which was not affected by a history of CXL or contact lens wear.

REVO-NX has previously been reported to have excellent repeatability in normal and cataractous eyes for AL, ACD, CCT, and LT [15, 26, 27]. In normal eyes, REVO-NX was found to have good agreement with IOLMaster700 (Carl Zeiss Meditec AG, Germany) [26], but poor agreement with optical low coherence reflectometer (Lenstar LS 900, Haag-Streit AG, Ohio, USA) [15], Scheimpflug imaging (Galilei G6, Ziemer, Port, Switzerland), and swept-source OCT (Casia, Tomey, Nagoya, Japan) [27].

In eyes with keratoconus, several studies have reported good repeatability in ACD, CCT, and TCT measurements using various AS-OCT and Scheimpflug imaging devices, including spectral-domain AS-OCT (Bioptigen Inc., Durham, North Carolina, USA and Optovue, California, USA) [11, 28], swept-source AS-OCT (CASIA SS1000, Tomey, Nagoya, Japan), Fourier-domain AS-OCT (Casia SS-1000, Tomey Corp, Nagoya, Japan) [15], Pentacam Scheimpflug imaging (Oculus Optikgerate GmbH, Wetzlar, Germany) [11, 28], and TMS-5 Scheimpflug imaging (Tomey, Erlangen, Germany) [29]. In keratoconus, most authors caution that AS-OCT and Scheimpflug imaging devices should not be used interchangeably for ACD, CCT, and TCT measurements [11, 13, 28, 29]. Some authors, however, have reported insignificant differences in ACD and CCT measurements yielded by AS-OCT and Scheimpflug imaging in keratoconus [14].

The results of this study indicate better repeatability using Scheimpflug imaging compared to AS-OCT in AL, ACD, CCT, and TCT. This is in contrast to previous studies, where Fourier-domain AS-OCT (Casia SS-1000, Tomey Corp, Nagoya, Japan) was more repeatable than Scheimpflug imaging (Pentacam HR, Oculus, Wetzlar, Germany) for CCT, TCT, and ACD in patients with keratoconus [13], while AS-OCT (CASIA SS-1000, Tomey, Nagoya, Japan) was more repeatable than Scheimpflug imaging (TMS-5, Tomey, Erlangen, Germany) for CCT in patients with keratoconus [29].

Contrary to the findings of this study, Yazici *et al*. found no significant difference in mean ACD and CCT in keratoconic eyes (Amsler-Krumeich Grades I-III) using time-domain OCT (Visante OCT, Carl Zeiss Meditec, California, USA), Placido disc-based Scanning Slit topography (Orbscan IIz, Bausch & Lomb, Rochester, NY, USA), and Scheimpflug imaging (Pentacam, Oculus, Lynnwood, WA, USA) [14]. Differences in the analysis method may have contributed to this discrepancy, where Yazici *et al*. compared absolute mean measurement values between devices, whereas we compared the mean difference between devices.

Measurement variation in AL and CCT had a statistically significant positive correlation with K_MAX_ in eyes with keratoconus. This is in keeping with the report from Hashemi *et al*., who found that a K_MAX_ of more than 55 Diopters resulted in lower repeatability using Scheimpflug imaging (Pentacam HR, Oculus, Wetzlar, Germany) [30].

The effect of disease severity on biometric repeatability is not well understood. Hashemi *et al*. found that ACD repeatability was not affected by disease severity using Orbscan II and Pentacam [31], but Flynn *et al*. reported reduced K_MAX_ repeatability with Scheimpflug imaging in higher Amsler-Krumeich grades of keratoconus [32]. Whilst no previous study has investigated the repeatability of AS-OCT derived biometric measurements in patients with severe keratoconus, the results of this study suggest that AS-OCT may provide more repeatable measurements in more severe keratoconus. Further research which directly compares AS-OCT to other devices in larger patient cohorts is necessary to confirm this.

CXL did not have a significant impact on the repeatability of measurements in eyes with keratoconus across AL, ACD, CCT, and LT using both REVO-NX and Pentacam-AXL. Hashemi *et al*. also found no statistically significant changes in repeatability in all anterior corneal indices before and 12 months after crosslinking using the Pentacam device [33]. As the effect of CXL on crosslinking is not well established, further longitudinal studies with larger numbers are required to confirm the accuracy of this observation, which is the other component of measurement accuracy [34].

We did not find any statistically significant differences in repeatability in eyes with a history of prior contact lens use. The effect of contact lens wear on the repeatability of biometric measurements in the context of keratoconus is poorly described. In healthy eyes, Lewis *et al*. found that repeatability of AL using IOLmaster (IOLMaster, Carl Zeiss Meditec AG, Jena, Germany) was not affected by the use of soft contact lenses [35].

Our study has several limitations. We did not have a healthy control group and we also included some scans of acceptable but not ideal quality, which we feel is a realistic representation of a clinical setting where patients with keratoconus often have scans of suboptimal quality. Moreover, contact lens wearers were only required to remove their contact lenses for 48 hours prior, however, a study has shown that soft contact lens, does not induce significant alteration in corneal shape or subsequent biometric measurements [36]. While this study has a modest number of participants, future studies with larger numbers across a variety of pathologies can help us to better understand the application of these devices in patients with keratoconus. Keratometric and IOL measurements were not reported as it was not an available feature on the REVO-NX software at the time of the study. The available data, however, could be employed in available keratometry devices to calculate the IOL [15].

In conclusion, REVO-NX and Pentacam-AXL exhibit good repeatability of biometric measurements in patients with keratoconus. Repeatability was higher with Pentacam-AXL, irrespective of a history of prior CXL or contact lens use. A higher K_MAX_ correlated with higher measurement variability, but repeatability was not significantly different between grades of disease severity. Interchangeable use of the two devices should, however, be avoided due to poor agreement.

## References

[1] Rabinowitz YS. Keratoconus. Surv Ophthalmol. 1998;42(4):297–319. 10.1016/s0039-6257(97)00119-7 9493273

[2] Ziaei M, Barsam A, Shamie N, Vroman D, Kim T, Donnenfeld ED, et al. Reshaping procedures for the surgical management of corneal ectasia. J Cataract Refract Surg. 2015;41(4):842–72. 10.1016/j.jcrs.2015.03.010 25840308

[3] Ziaei M, Gokul A, Vellara H, et al. Prospective two-year study of clinical outcomes following epithelium-off pulsed versus continuous accelerated corneal crosslinking for keratoconus. Clin Exp Ophthalmol 2019;33:1897–1903. 10.1111/ceo.13567 31170327

[4] Ziaei M, Vellara H, Gokul A, et al. Prospective 2-year study of accelerated pulsed transepithelial corneal crosslinking outcomes for Keratoconus. Eye (Lond) 2019; 47:980–986. 10.1038/s41433-019-0502-3 31273313 PMC7002515

[5] Keane M, Coster D, Ziaei M, et al. Deep anterior lamellar keratoplasty versus penetrating keratoplasty for treating keratoconus. Cochrane Database Syst Rev. 2014 7 22;(7):CD009700. 10.1002/14651858.CD009700.pub2 25055058 PMC10714035

[6] Ziaei M, Vellara HR, Gokul A, et al. Comparison of corneal biomechanical properties following penetrating keratoplasty and deep anterior lamellar keratoplasty for keratoconus. Clin Exp Ophthalmol. 2020 3;48(2):174–182. 10.1111/ceo.13677 31705767

[7] Ziaei M, Sharif-Paghaleh E, Manzouri B. Pharmacotherapy of corneal transplantation. Expert Opin Pharmacother. 2012 4;13(6): 829–40. 10.1517/14656566.2012.673588 22424532

[8] Thebpatiphat N, Hammersmith KM, Rapuano CJ, Ayres BD, Cohen EJ. Cataract surgery in keratoconus. Eye Contact Lens. 2007;33(5):244–6. 10.1097/ICL.0b013e318030c96d 17873627

[9] Kane JX, Connell B, Yip H, McAlister JC, Beckingsale P, Snibson GR, et al. Accuracy of Intraocular Lens Power Formulas Modified for Patients with Keratoconus. Ophthalmology. 2020;127(8):1037–42. 10.1016/j.ophtha.2020.02.008 32279887

[10] Ghiasian L, Abolfathzadeh N, Manafi N, Hadavandkhani A. Intraocular lens power calculation in keratoconus; A review of literature. J Curr Ophthalmol. 2019;31(2):127–34. 10.1016/j.joco.2019.01.011 31317089 PMC6611933

[11] Kumar M, Shetty R, Jayadev C, Dutta D. Comparability and repeatability of pachymetry in keratoconus using four noncontact techniques. Indian journal of ophthalmology. 2015;63(9):722–7. 10.4103/0301-4738.170987 26632128 PMC4705708

[12] Shetty R, Arora V, Jayadev C, Nuijts RM, Kumar M, Puttaiah NK, et al. Repeatability and agreement of three Scheimpflug-based imaging systems for measuring anterior segment parameters in keratoconus. Invest Ophthalmol Vis Sci. 2014;55(8):5263–8. 10.1167/iovs.14-15055 25074774

[13] Szalai E, Berta A, Hassan Z, Modis L Jr. Reliability and repeatability of swept-source Fourier-domain optical coherence tomography and Scheimpflug imaging in keratoconus. J Cataract Refract Surg. 2012;38(3):485–94. 10.1016/j.jcrs.2011.10.027 22261325

[14] Yazici AT, Pekel G, Bozkurt E, Yildirim Y, Pekel E, Demirok A, et al. Measurements of anterior segment parameters using three different non-contact optical devices in keratoconus patients. Int J Ophthalmol. 2013;6(4):521–5. 10.3980/j.issn.2222-3959.2013.04.21 23991390 PMC3755315

[15] Kanclerz P, Hoffer KJ, Rozema JJ, Przewłócka K, Savini G. Repeatability and reproducibility of optical biometry implemented in a new optical coherence tomographer and comparison with a optical low-coherence reflectometer. Journal of Cataract & Refractive Surgery. 2019;45(11):1619–24. 31706516 10.1016/j.jcrs.2019.07.002

[16] Muzyka-Woźniak M, Oleszko A. Comparison of anterior segment parameters and axial length measurements performed on a Scheimpflug device with biometry function and a reference optical biometer. International Ophthalmology. 2019;39(5):1115–22. 10.1007/s10792-018-0927-x 29700651

[17] Zadnik K, Barr JT, Edrington TB, Everett DF, Jameson M, McMahon TT, et al. Baseline findings in the Collaborative Longitudinal Evaluation of Keratoconus (CLEK) Study. Invest Ophthalmol Vis Sci. 1998;39(13):2537–46. 9856763

[18] Nordan LT. Keratoconus: diagnosis and treatment. Int Ophthalmol Clin. 1997;37(1):51–63. 10.1097/00004397-199703710-00005 9101345

[19] Belin MW, Duncan JK. Keratoconus: The ABCD Grading System. Klin Monbl Augenheilkd. 2016;233(6):701–7. 10.1055/s-0042-100626 26789119

[20] Goebels S, Eppig T, Wagenpfeil S, Cayless A, Seitz B, Langenbucher A. Staging of keratoconus indices regarding tomography, topography, and biomechanical measurements. Am J Ophthalmol. 2015;159(4):733–8. 10.1016/j.ajo.2015.01.014 25634534

[21] Feng Y, Varikooty J, Simpson TL. Diurnal variation of corneal and corneal epithelial thickness measured using optical coherence tomography. Cornea. 2001;20(5):480–3. 10.1097/00003226-200107000-00008 11413402

[22] Bland JM, Altman DG. Statistical methods for assessing agreement between two methods of clinical measurement. Lancet. 1986;1(8476):307–10. 2868172

[23] Wang W, Miao Y, Savini G, McAlinden C, Chen H, Hu Q, et al. Precision of a new ocular biometer in eyes with cataract using swept source optical coherence tomography combined with Placido-disk corneal topography. Sci Rep. 2017;7(1):13736. 10.1038/s41598-017-13800-7 29061989 PMC5653855

[24] Shankar H, Taranath D, Santhirathelagan CT, Pesudovs K. Anterior segment biometry with the Pentacam: comprehensive assessment of repeatability of automated measurements. J Cataract Refract Surg. 2008;34(1):103–13. 10.1016/j.jcrs.2007.09.013 18165089

[25] Ernst BJ, Hsu HY. Keratoconus association with axial myopia: a prospective biometric study. Eye Contact Lens. 2011;37(1):2–5. 10.1097/ICL.0b013e3181fb2119 21178694

[26] Sikorski BL, Suchon P. OCT Biometry (B-OCT): A New Method for Measuring Ocular Axial Dimensions. J Ophthalmol. 2019;2019:9192456. 10.1155/2019/9192456 31511790 PMC6710804

[27] Wylęgała A, Mazur R, Bolek B, Wylęgała E. Reproducibility, and repeatability of corneal topography measured by Revo NX, Galilei G6 and Casia 2 in normal eyes. PLoS One. 2020;15(4):e0230589. 10.1371/journal.pone.0230589 32240192 PMC7117679

[28] Grewal DS, Brar GS, Grewal SP. Assessment of central corneal thickness in normal, keratoconus, and post-laser in situ keratomileusis eyes using Scheimpflug imaging, spectral domain optical coherence tomography, and ultrasound pachymetry. J Cataract Refract Surg. 2010;36(6):954–64. 10.1016/j.jcrs.2009.12.033 20494767

[29] Chan TCY, Biswas S, Yu M, Jhanji V. Comparison of corneal measurements in keratoconus using swept-source optical coherence tomography and combined Placido-Scheimpflug imaging. Acta Ophthalmol. 2017;95(6):e486–e94. 10.1111/aos.13298 27805316

[30] Hashemi H, Yekta A, Khabazkhoob M. Effect of keratoconus grades on repeatability of keratometry readings: Comparison of 5 devices. J Cataract Refract Surg. 2015;41(5):1065–72. 10.1016/j.jcrs.2014.08.043 26049838

[31] Hashemi H, Asharlous A, Aghazadeh Amiri M, Yekta A, Ramin S, Taheri A, et al. Intrasubject Repeatability and Interdevice Agreement of Anterior Chamber Depth Measurements by Orbscan and Pentacam in Different Grades of Keratoconus. Eye Contact Lens. 2019;45(1):51–4. 10.1097/ICL.0000000000000515 29944509

[32] Flynn TH, Sharma DP, Bunce C, Wilkins MR. Differential precision of corneal Pentacam HR measurements in early and advanced keratoconus. Br J Ophthalmol. 2016;100(9):1183–7. 10.1136/bjophthalmol-2015-307201 26659714

[33] Hashemi H, Mehravaran S, Asgari S. The effect of corneal cross-linking on the anterior and posterior parameters of the cornea: A prospective repeatability study. Rom J Ophthalmol. 2019;63(1):68–74. 31198900 PMC6531775

[34] McAlinden C, Khadka J, Pesudovs K. Precision (repeatability and reproducibility) studies and sample-size calculation. J Cataract Refract Surg. 2015;41(12):2598–604. 10.1016/j.jcrs.2015.06.029 26796439

[35] Lewis JR, Knellinger AE, Mahmoud AM, Mauger TF. Effect of soft contact lenses on optical measurements of axial length and keratometry for biometry in eyes with corneal irregularities. Invest Ophthalmol Vis Sci. 2008;49(8):3371–8. 10.1167/iovs.07-1247 18441314

[36] Goudie C, Tatham A, Davies R, Sifton A, Wright M. The effect of the timing of the cessation of contact lens use on the results of biometry. Eye (Lond). 2018;32(6):1048–54. 10.1038/s41433-018-0019-1 29391575 PMC5997671

