# Correction: Repeatability and agreement of biometric measurements using spectral domain anterior segment optical coherence tomography and Scheimpflug tomography in keratoconus

**DOI:** 10.1371/journal.pone.0314484

**Published:** 2024-11-21

**Authors:** Ye Li, Akilesh Gokul, Charles McGhee, Mohammed Ziaei

In Table 2, the data listed in the precison and repeatability column of Pentacam-AXL under TCT (μm) Group A is incorrect. The correct precision is 5.84 and the repeatability is 8.26. Also, the data listed in the ICC 95% Confidence Interval column of Revo NX under CCT (μm) Group A should be 0.97 to 0.99. Please see the correct Table 2 here.

**Table 2:** Sw, precision, repeatability, CV, and ICC (95% confidence interval).

Parameter (units)	Mean ± SD	Within-Subject SD	Precision	Repeatability	CV (%)	ICC	ICC 95% Confidence Interval
**GROUP A (n = 95)**							
ACD (mm)							
- REVO NX	3.75 ± 0.31	0.08	0.16	0.22	0.82	0.83	0.75 to 0.88
- Pentacam-AXL	3.83 ± 0.31	0.03	0.05	0.08	0.40	0.99	0.99 to 1.00
CCT (μm)							
- REVO NX	480.28 ± 42.25	10.73	21.03	29.72	1.59	0.98	0.97 to 0.99
- Pentacam-AXL	474.89 ± 42.44	2.92	5.73	8.10	0.43	0.99	0.99 to 1.00
AL (mm)							
- REVO NX	24.02 ± 0.85	0.13	0.25	0.35	0.09	0.99	0.99 to 1.00
- Pentacam-AXL	23.97 ± 0.83	0.01	0.03	0.04	0.00	1.00	1.00 to 1.00
TCT (μm)							
- REVO NX	440.19 ± 46.54	13.25	25.96	36.69	1.01	0.97	0.96 to 0.98
- Pentacam-AXL	464.84± 44.65	2.98	5.84	8.26	0.44	0.99	0.99 to 0.99
LT (mm)							
- REVO NX	4.89 ± 12.64	0.08	0.16	0.22	1.28	0.98	0.97 to 0.98
**GROUP B (n = 86)**							
ACD (mm)							
- REVO NX	3.80±0.33	0.10	0.19	0.27	1.08	0.97	0.96 to 0.98
- Pentacam-AXL	3.89±0.35	0.03	0.06	0.08	0.43	0.99	0.99 to 1.00
CCT (μm)							
- REVO NX	466.44±43.66	9.65	18.91	26.72	1.35	0.98	0.98 to 0.99
- Pentacam-AXL	456.26±45.18	3.70	7.26	10.26	0.49	0.99	0.99 to 1.00
AL(mm)							
- REVO NX	23.91±0.95	0.08	0.16	0.23	0.05	0.99	0.99 to 1.00
- Pentacam-AXL	23.89±0.96	0.02	0.04	0.06	0.00	1.00	1.00 to 1.00
TCT (μm)							
- REVO NX	424.99 ± 43.47	5.18	10.15	14.35	0.56	0.99	0.99 to 1.00
- Pentacam-AXL	444.81 ± 45.13	3.48	6.82	9.63	0.62	0.99	0.99 to 1.00
LT (mm)							
- REVO NX	3.57±0.27	0.15	0.29	0.42	1.52	0.90	0.85 to 0.93
**GROUP C (n = 33)**							
ACD (mm)							
- REVO NX	3.70±0.38	0.03	0.06	0.09	0.70	0.99	0.99 to 1.00
- Pentacam-AXL	3.79±0.39	0.03	0.05	0.07	0.39	0.99	0.99 to 1.00
CCT(μm)							
- REVO NX	450.26±43.04	6.49	12.71	17.97	1.03	0.99	0.99 to 1.00
- Pentacam-AXL	444.43±47.89	4.48	8.77	12.40	0.58	0.99	0.99 to 1.00
AL (mm)							
- REVO NX	23.95±0.98	0.10	0.20	0.28	0.06	0.99	0.99 to 1.00
- Pentacam-AXL	23.92±0.97	0.01	0.03	0.04	0.00	1.00	1.00 to 1.00
TCT (μm)							
- REVO NX	416.33±45.59	3.25	6.37	9.00	0.27	0.99	0.99 to 1.00
- Pentacam-AXL	435.17±45.28	4.33	8.49	11.99	0.61	0.99	0.99 to 1.00
LT (mm)							
- REVO NX	3.61±0.33	0.06	0.12	0.17	1.21	0.99	0.98 to 0.99

CV, coefficient of variation; ICC, intraclass correlation coefficient; ACD, anterior chamber depth; CCT, central corneal thickness; AL, axial length; TCT, thinnest corneal thickness; LT, lens thickness

## References

[1] Li Y, Gokul A, McGhee C, Ziaei M (2021) Repeatability and agreement of biometric measurements using spectral domain anterior segment optical coherence tomography and Scheimpflug tomography in keratoconus. PLoS ONE 16(5): e0248659. doi: 10.1371/journal.pone.0248659 34019547 PMC8139453

